# The phenomenon of autonomous endosperm in sexual and apomictic plants

**DOI:** 10.1093/jxb/erad168

**Published:** 2023-05-08

**Authors:** Joanna Rojek, Nir Ohad

**Affiliations:** Department of Plant Cytology and Embryology, Faculty of Biology, University of Gdansk, Gdansk, Poland; School of Plant Sciences and Food Security, Tel Aviv University, Tel Aviv, Israel; John Innes Centre, UK

**Keywords:** Apomixis, autonomous endosperm, autonomous seed, auxin, endosperm development, FIS-POLYCOMB, mammalian sex hormones, parthenogenesis, plant female gametophyte, plant reproduction

## Abstract

Endosperm is a key nutritive tissue that supports the developing embryo or seedling, and serves as a major nutritional source for human and livestock feed. In sexually-reproducing flowering plants, it generally develops after fertilization. However, autonomous endosperm (AE) formation (i.e. independent of fertilization) is also possible. Recent findings of AE loci/ genes and aberrant imprinting in native apomicts, together with a successful initiation of parthenogenesis in rice and lettuce, have enhanced our understanding of the mechanisms bridging sexual and apomictic seed formation. However, the mechanisms driving AE development are not well understood. This review presents novel aspects related to AE development in sexual and asexual plants underlying stress conditions as the primary trigger for AE. Both application of hormones to unfertilized ovules and mutations that impair epigenetic regulation lead to AE development in sexual *Arabidopsis thaliana*, which may point to a common pathway for both phenomena. Apomictic-like AE development under experimental conditions can take place due to auxin-dependent gene expression and/or DNA methylation.

## Teaser

Understanding the mechanisms of fertilization-independent, autonomous endosperm (AE) formation in sexually-reproducing plants will enable the regulation of such an autonomous pathway in fertilization-dependent crops.

## Introduction

**Fig. 1.**
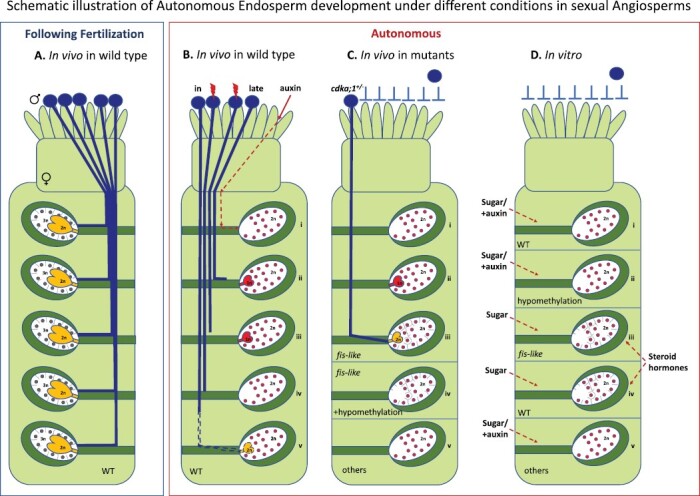
Schematic illustration of AE development under different conditions in sexual angiosperms. (A) In angiosperms, endosperm develops following successful pollination and fertilization. In the common Polygonum-type embryo sac, such as in sexual Arabidopsis, the pollen tube carries two sperm cells which participate in a double fertilization process. First, the haploid egg cell (♀1n) is fertilized by a haploid sperm (♂1n) to form a diploid embryo (2n). The second haploid sperm arriving with the same pollen tube fertilizes the homodiploid central cell to form a biparental 3n endosperm. (B-D) Endosperm can also develop autonomously (AE), i.e. independently from fertilization under different conditions. In such cases, AE will have a 2n content as opposed to 3n when the endosperm results from fertilization of the central cell. (B) AE can be induced *in vivo* under different experimental conditions. (B.i) Following emasculation and the addition of exogenous auxin application is sufficient to trigger multinuclear AE development. (B.ii) AE formation can be induced after delayed or interspecific and intergeneric pollination (named ‘late’), in which case both embryo and endosperm are formed. (B.iii–iv) Irradiation of pollen interrupts or inhibits pollen tube growth and pollen viability, yet allows autonomous seed formation; in autonomous seeds either both embryo (via parthenogenesis) and AE can develop (iii), or only AE (iv). (B.v) AE may result from pollination with incompatible pollen (in); in other cases, the sperm may fertilize the egg cell, whereas the central cell remains unfertilized, yet AE develops through a limited round of divisions. (C) Mutation in *Polycomb Group Protein* (*PcG*) genes, which have a significant role in the regulation of endosperm development, leads to initiation of AE development in Arabidopsis ovules in the absence of fertilization, presenting a ‘*fis*-like’ phenotype (e.g. Ohad *et al.*, 1996, 1999; Kiyosue *et al.*, 1999), where AE has a 2n content. AE formed in *fis-PRC2* mutants displays limited development (AE cellularization is rare in *medea* and *fis2* mutants; Chaudhury *et al.*, 1997; Grossniklaus *et al.*, 1998). Most often AE develops only until the multinuclear stage (C.i), unable to proceed to the next stage—cellularization; a parthenogenetic underdeveloped embryo may accompany the AE (C.ii). In the case of pollination by *cdka;1* mutant pollen (Nowack *et al.*, 2006, 2007), which carries only a single haploid gamete, it fertilizes the egg cell, leaving the central cell unfertilized. In such ovules, the central cell autonomously undergoes free nuclear divisions or even cellularization with similar characteristics of WT endosperm. In such cases the developing AE is sufficient to sustain complete seed development (C.iii). The *fis*-like phenotype is also complemented in the *fie* mutant with a low-methylated genome (*fie-1*/*FIE*; *MET1* a/s; Ungru *et al.*, 2008) in which endosperm development proceeds further and cellularizes in the absence of fertilization (C.iv). In other mutants (‘others’, C.v), such as *rbr1 (retinoblastoma related 1*; Guitton *et al.*, 2004), and mutants with altered auxin signalling: *rgtb1* (*RAB geranylgeranyl transferase beta-subunit 1*; Rojek *et al.,* 2021b) or *DD65::TAA1; DD65::YUC6* (Figueiredo *et al.*, 2015) AE usually is underdeveloped (multinuclear stage). (D.i-v.) *In vitro* culture of unfertilized ovules (inside unpollinated ovary) may trigger AE development under certain conditions. A basic medium enriched with a higher (5–10%) sucrose or glucose concentration is sufficient to initiate AE. However, higher efficiency of AE development is usually achieved in the presence of auxin (D.i,v) and with along with hypomethylation (via mutation or chemical treatment; (D.ii). In addition, epibrassinolide and mammalian sex hormones evidently improve the frequency of AE in *fie-1* mutant ovules, and are sufficient to allow the development of mature endosperm (cellular endosperm), at least in *A. thaliana* (D.iii-iv). C.v ‘others’ also represent cases of AE induction under additional/different culture conditions, e.g. cytokinin, higher temperature, osmotic pressure, starvation (see Table 4 for details; Mól *et al.*, 1995; Wijowska *et al.*, 1999b; Rojek *et al.*, 2013).

One of the major challenges facing our society is to feed approximately 9 billion people without exerting huge pressure on the planet. This viewpoint is reflected in popular scientific magazines, journals, and global summits (e.g. https://www.nationalgeographic.com/foodfeatures/feeding-9-billion/; https://www.canada.ca/en/services/environment/wildlife-plants-species/biodiversity/cop15.html). Various approaches have been being developed to address this food dilemma; however, almost all of these efforts focus on agriculture and crop seeds. In fact, a major part of the nutrition within the seed (i.e. the fertilized ovule) is derived from the endosperm. The ovule contains a female gametophyte (embryo sac), which is the donor of two gametes: the egg cell and the central cell (Sprunck and Gross-Hardt, 2011). Following double fertilization events, the egg cell develops an embryo, and the central cell initiates the development of the endosperm (Fig. 1A). Besides leading the communication and coordination of distinct genetic programmes that control the development of each seed component, the central role of the endosperm is to nourish the embryo (e.g. Li and Berger, 2012; Lafon-Placette and Köhler, 2014) and the developing seedling (Chahtane *et al.*, 2017).

Proper development of the endosperm relies on the balance between maternal and paternal chromosomal contribution (Endosperm Balance Number = EBN; 2:1 maternal-to-paternal genome ratio; 2m:1p) and complex control of the maternal and paternal alleles, i.e. parent of origin effects (e.g. Scott *et al.*, 1998).

EBN applies to the majority of flowering plants, including most of the apomicts in which meiosis and fertilization of the egg cell are bypassed yet central cell fertilization is required. However, some populations or species do not follow this rule and produce viable seeds with other than 2m:1p EBN (Köhler *et al.*, 2010; Dziasek *et al.*, 2021; Paczesniak *et al.*, 2022). The deviation from 2m:1p EBN is particularly pronounced in apomictic plants, where in extreme cases the ratio of ≥2m:0p allows the seed to develop to maturity, independently of the male genome (autonomous apomicts; e.g. Bicknell and Koltunow, 2004). Endosperm with 2m:0p EBN can also develop in native sexual plants, although this phenomenon seems to be rare (Table 1). Thus, endosperm can develop independently from fertilization, resulting in an autonomous endosperm (AE). AE can occur in reduced (after meiosis) and unreduced (omitting meiosis) female gametophytes.

**Table 1.** Occurrence of AE in sexual angiosperms *in vivo*/*in planta*

Taxon	Population or genotype	Experimental regime	Method	Added factor(s)	Embryo companion	Occurrence (frequency)	Advancement in the development	References
	***In vivo*/ *in planta***							
*Anemone nemorosa*	Clonal populations from Poland and France	Emasculation; interclonal crossing	Chromosome counting; embryological analysis; cytophotometry	Self-incompatibility	2–4 celled embryo development after single fertilization	~100% in tested flowers	Up to 64 nuclei of AE	Trela (1963a, b); Brouland (1968); Trzcińska (2007)
*Anemone ranunculoides*	Clonal populations from Poland and France	Emasculation; interclonal crossing	Chromosome counting; embryological analysis	Self-incompatibility	2–4 celled embryo development after single fertilization	~100% in tested flowers	Up to 64 nuclei of AE	Trela-Sawicka (1974)
*Arabidopsis thaliana*	WT ecotype; WT cultivar Col-0	Emasculation and prevention from pollination	Embryological analysis; auxin detection	None	No	~2.8–4%	Few to several nuclei of AE	Figueiredo *et al.* (2015); Rojek *et al.* (2021b)
*Juglans regia*	Monoecious, dichogamous, protandrous cultivar	Prevention from pollination	Gibberellin content measurement; embryological analysis	N/a	No	In the majority of unpollinated flowers	Precocious cellularization in several-nuclear AE	Tadeo *et al.* (1994)
*Lycopersicon esculentum*	Cultivar	Prevention from pollination	Embryological analysis	N/a	No	Several ovules in one among five genotypes tested	Multinuclear AE	Adamowicz *et al.* (2000)
*Zea mays*	2x KZR136-1 line	Prevention from pollination	Chromosome counting; embryological analysis	N/a	No	N/a	Polar nuclei fused without fertilization. Starch-less endosperm at various stages	Laikova (1976)

N/a—not applicable or not available.

Available data show that the unfertilized central cell has the potential to develop into an endosperm, but this potential is suppressed until fertilization takes place. Both suppression and activation of the central cell to initiate endosperm development are controlled by several mechanisms recruiting the Polycomb Repressive Complex 2 (PRC2), transcription factors, and hormonal regulators (reviewed in Hands *et al.*, 2016). AE development was observed in sexual plants carrying mutations affecting the *Polycomb* genes (*PcG*) (e.g. Ohad *et al.*, 1996, 1999; Chaudhury *et al.*, 1997; Kiyosue *et al.*, 1999; Luo *et al.*, 1999; Guitton *et al.*, 2004). Additionally, auxin, a main hormonal regulator for seed development after fertilization, can trigger AE formation when it is ectopically expressed in the central cell and the sporophyte tissue of the unfertilized ovule. Recent studies have focused on the initiation of AE development in *Arabidopsis thaliana* FERTILIZATION INDEPENDENT SEED (FIS)-class mutants (e.g. Kordyum and Mosyakin, 2020) and in response to elevated auxin levels in the underdeveloped central cell (Figueiredo *et al.*, 2015; Figueiredo and Köhler, 2018). Furthermore, fully developed AE was reported to be produced in *Hieracium* lines where AE formation took place within the meiotically derived embryo sac (*AutE*; Ogawa *et al.*, 2013).

An important issue that is not yet fully understood is the mutual dependence of the development of the embryo and endosperm on each other. Both the embryo and the endosperm can start developing independently from one another, as has been shown in mutants created with a single sperm cell delivered to the female gametophyte (e.g. *cdka;1*, Nowack *et al.*, 2007; Ungru *et al.*, 2008; *dmp8 dmp9*, Xiong *et al.*, 2021; Chen *et al.*, 2022). In this context, the term ‘autonomous’ implies the development of these structures independently from fertilization signals (Zhang, 2021). Although some studies describe at least in part the autonomous development of an embryo, further embryo growth depends on the presence of the fully developed endosperm (Ungru *et al.*, 2008).

This review discusses AE formation in the context of cyto-embryological and molecular research, focusing on Arabidopsis and closely related genera. Special emphasis is placed on AE induction and development in sexual plants under experimental (*in vitro*) conditions.

To our knowledge, at least 31 species of wild-type (WT) sexual flowering plants that can develop AE have been reported (Tables 1–4; Fig. 1). In the case of sexual Arabidopsis, AE is easily induced in WT plants under appropriate experimental stress conditions [e.g. higher sucrose concentration (Rojek *et al.*, 2005), external supplementation of hormones (Rojek *et al.*, 2013, 2015; Figueiredo *et al.*, 2015), or demethylating agents (Rojek *et al.*, 2013, 2015)]. AE can fully develop *in vitro* (i.e. cellularization takes place; Rojek *et al.*, 2015) unlike the AE in *FIS*-class mutants that remain underdeveloped. Several reports provide evidence that the apomictic pathway can be replaced by the sexual one, and vice versa, in response to stress *in planta* and *in vitro*; thus, AE in such cases may develop as a result of a stress response when ovules trigger sexual or apomictic pathways by altering homeostasis-based processes of stress perception and attenuation (Carman *et al.*, 2011; Horandl and Hadacek, 2013; Mateo de Arias *et al.*, 2020).

## Circumstances leading to AE formation

### Development of nutritive tissue in gymnosperms and angiosperms

The evolution of endosperm in angiosperms and its relationship to the evolution of nutritive tissue in gymnosperms has been widely discussed (e.g. Costa *et al.*, 2004; Kordyum and Mosyakin, 2020; Li and Yang, 2020). One of the hypotheses regarding the origin of endosperm suggests that it is homologous to a gymnosperm female gametophyte that became fertilization dependent. This scenario is possible as endosperm development in angiosperms is as proliferative as that of the gymnosperm female gametophyte, and the input of a paternal genome to the female precursor central cell may lead to hybrid vigour, which may allow for biparental control over resource allocation to the embryo (Baroux *et al.*, 2002; reviewed in Kordyum and Mosyakin, 2020).

In gymnosperms, the large haploid female gametophyte nourishes the embryo after fertilization, whereas in sexual angiosperms, this role has been adopted by the endosperm that forms after fertilization, which accompanies the developing embryo (Williams and Friedman, 2002). Though most of the angiosperms have a Polygonum-type embryo sac containing two polar nuclei that produce a triploid (3n) endosperm, a diploid (2n) endosperm originating from a central cell with only one polar nucleus has been described in several families of basal angiosperms (reviewed in Baroux *et al.*, 2002). A study of the endosperm in basal flowering plants such as the waterlily family (*Nymphaeaceae*) suggests that their diploid endosperm may represent an ancestral angiosperm condition (Williams and Friedman, 2002). From this perspective, the megagametophyte tissue of gymnosperms functionally plays an equivalent role to that of the endosperm in angiosperms, although it is haploid and fertilization-independent as is the homodiploid AE found in angiosperms.

Recently, Qiu and Köhler (2022) have proposed that the duplication and diversification of Type I MADS-box transcription factors (MTFs) underpin the evolution of the endosperm. MTFs are an evolutionary ancient class and major developmental regulators (Alvarez-Buylla *et al.*, 2000).

Three major clades of MADS-box genes, M*α*, M*β*, and M*γ* are demonstrated in seed plants. In gymnosperms, ancestral M*α* and M*β*-like likely dimerize and function in maternal tissues. Angiosperms express M*α* and M*β* heterodimers in maternal tissues, whereas M*γ* genes have undergone neofunctionalization for an endosperm-specific function, probably enabling endosperm development.

Apart from the nourishing function, the endosperm is a source of epigenetic processes in which PRC2 is particularly important [e.g. Batista and Köhler (2020) and references cited therein], for example, FIS-PRC2 (repressive complex) prevents AE formation and thus couples fertilization with endosperm development (Guitton and Berger, 2005). In gymnosperms, where the female gametophyte forms an AE-like nourishing structure, a FIS-PRC2-like complex would not be required (according to Köhler and Lafon-Placette, 2015).

### Occasional AE development in native apomicts

Although not common, AE is primarily associated with apomixis (Kirioukhova *et al.*, 2018; Rojek *et al.*, 2018; Albertini *et al.*, 2019; Carman *et al.*, 2019; Fei *et al.*, 2019; Hojsgaard and Hörandl, 2019; León-and Vielle-Calzada, 2019; Schmidt, 2020; Chen *et al.*, 2022; Vernet *et al.*, 2022). The three necessary developmental components of apomixis are (i) the formation of unreduced gametes (apomeiosis), (ii) embryogenesis free from fertilization (parthenogenesis) and (iii) functional endosperm production—autonomously or through pseudogamy (e.g. Mogie, 1992; Curtis and Grossniklaus, 2008).

*Sensu stricto*, in many species the endosperm is absorbed by the embryo through programmed cell death (PCD) (Buono *et al.*, 2019), whereas the embryo further develops in the developing seed.

Therefore, all these phenomena—parent-of-origin effects, genomic dosage in triploid tissue, epigenetic mechanisms/ imprinting, and selection for mutations beneficial to endosperm development ‘carried forward’ by the zygote—are temporal (see, e.g. Baroux and Grossniklaus, 2019; Lafon-Placette, 2020). However, the products of imprinted genes and epigenetic control in the central cell and then in the endosperm (sRNA, RNAi, methylation status, etc.) directly affect the egg cell and the developing embryo, respectively (see reviews, e.g. Köhler *et al.*, 2012; Gehring and Satyaki, 2017). In another context, although endosperm is finally consumed by the embryo or seedling and disappears prior to the formation of the gametes of the next generation, the characteristics of the endosperm can be stored in the embryo for the next generation epigenetically. This way, the features of central cell/ endosperm are inherited (e.g. maternal *fie* mutation that prevents the correct embryo formation; endosperm-based hybridization barriers that prevent interspecific crossing; see Ohad *et al.*, 1996; Lafon-Placette and Köhler, 2016). Furthermore, without the endosperm as a nutritive tissue, the embryo will abort (Lafon-Placette and Köhler, 2014; Hands *et al.*, 2016). Consequently, most apomicts require fertilization of the central cell for seed production (pseudogamous apomicts; Hörandl *et al.*, 2008, and references cited therein; Van Dijk *et al.*, 2020; Paczesniak *et al.*, 2022).

The formation of endosperm independently from fertilization is predominant in apomicts within the *Asteraceae* family and is often associated with polyploidy (Noyes, 2007; Van Dijk *et al.*, 2020). In *Taraxacum officinale*, AE is regularly present without parthenogenesis, indicating that the parthenogenesis locus does not control endosperm formation; instead modifier genes are required for AE development (Van Dijk *et al.*, 2020; Mau *et al.*, 2021). Another example is *Hieracium*, which comprises both sexual (diploids) and apomictic species/ populations (triploids, tetraploids), in which the endosperm develops independently of fertilization (Koltunow and Grossniklaus, 2003). Interestingly, though autonomous apomicts in *Erigeron* (*Asteraceae*) are mainly polyploid, diploid apomicts with AE are rarely observed (Noyes and Wagner, 2014).

The genus *Boechera* serves as an interesting test case for understanding AE development in which sexual reproduction occurs together with apomixis at various levels (diploid, triploid, tetraploid; Rojek *et al.*, 2018; Carman *et al.*, 2019; Mau *et al.*, 2021). Diploid *Boechera* sp. exhibit highly variable modes of seed formation, from obligate sexuality, through various levels of sexual and facultative apomictic seed, to obligate apomixis (reviewed in Rojek *et al.*, 2018). In sexual and apomictic species (or populations) of *Boechera*, endosperm development for the most part requires central cell fertilization (Aliyu *et al.*, 2010; Paczesniak *et al.*, 2022). However, both diploid and triploid apomictic *Boechera* produce a variety of endosperm ploidies, with examples exhibiting characteristics of AE (2:4, 3:6, 4:8, embryo:endosperm C DNA value; based on flow cytometry seed screening analysis; Aliyu *et al.*, 2010; Voigt-Zielinski *et al.*, 2012; Mau *et al.*, 2021; Paczesniak *et al.*, 2022). Moreover, AE and sexual endosperm development can take place in the same population or even in individual flowers (Roy, 1995; Aliyu *et al.*, 2010; Rojek *et al.*, 2018). Thus, AE can form in facultative apomicts, although the underlying causes of variation are complex, e.g. as a surplus effect of crossing between sexual and apomictic individuals, polyploidization, and mutation accumulation, followed by the loss of function for the need for the EBN. In *Boechera*, the widespread occurrence of ‘unbalanced’ diploid apomicts (after fertilization of an unreduced central cell by the haploid sperm cell) with pentaploid endosperm (4m:1p) supports the idea that strict maintenance of the EBN is not always necessary for proper seed formation (Mau *et al.*, 2021; Paczesniak *et al.*, 2022).

### AE development in sexual plants

In sexual plants, AE is formed from an unfertilized central cell when its homodiploid nucleus (i.e. after the fusion of two haploid polar nuclei) divides mitotically, leading to the formation of at least a binuclear cell. AE development can be interpreted as a disruption of normal fertilization. For example, in *A. thaliana*, several mutants manifest AE following failed fertilization or genetic alterations within the female gametophyte in the ovule. In such cases, a seed-like structure develops, containing only the endosperm, but not an embryo (e.g. Ohad *et al.*, 1996, 1999; Vinkenoog *et al.*, 2000; Figueiredo *et al.*, 2015; Rojek *et al.*, 2021b; Chen *et al.*, 2022).

An explanation for AE development from the central cell in sexual plants is still under debate, although this phenomenon has been observed since the beginning of the twentieth century (Shibata, 1902). Interest in AE in sexual plants gained an impetus when *in vitro* techniques were developed, and the phenomenon could be studied independently from external environmental cues. Although AE in sexual plants is considered a rare phenomenon, it is important to note that regarding apomicts, in some populations or species AE is common, in others it occurs sporadically. *A. thaliana* is an example of a species in which AE has been repeatedly reported, in several independent studies, in both *in planta* and *in vitro* experiments, and is studied by various methods. The ‘genetic cassette’ that triggers the development of AE in both sexual and apomictic plants remains to be uncovered, although it is already partially recognized to be specific, at least to the genus level (Mau *et al.*, 2021; Van Dijk *et al.*, 2020).

AE development under different conditions in sexual angiosperms is represented in Fig. 1. In Tables 1–4, a wide range of reports on AE from 31 taxa in which AE development occurs at least by embryological analysis are presented, which is a useful method for studying AE. Two exceptions have been made. In the case of *Theobroma cacao* (Falque, 1994; Table 2), AE has been cited for years, although the authors discussed the possibility that the same plant can produce both autonomous and amphimictic endosperm. In the case of the *bga-1/BGA* (*borgia*) mutant (Table 3), AE was determined on the basis of the ovule size; however, the assignment of mutation to the *fis*-class makes the statement highly likely.

**Table 2.** Occurrence of AE in sexual angiosperms in planta under experimental conditions.

Taxon	Population or genotype	Experimental regime	Method	Added factor(s)	Embryo companion	Occurrence (frequency)	Advancement in the development	References
	***In planta* under experimental conditions**							
*Actinidia deliciosa*	Male and female cultivars	Pollination with γ-irradiated pollen	Embryological analysis	Incomplete or failed transmission of the male genome	Few-celled to torpedo-shaped parthenogenetic embryos	15.9–25.6% of the ovules	Cellular-type AE (a feature of the species) but contained very low amount of storage products and smaller than normal endosperm	Musiał and Przywara (1998, 1999b)
*Arabidopsis thaliana*	WT Col-0	Emasculation; exogenous auxin application	Embryological analysis	2,4-D or IAA treatment	No	Up to 60% of the ovules	Several or multinuclear AE	Figueiredo *et al.* (2015)
*Brassica oleracea*	Cultivar	Prickled pollination	Embryological analysis	Prickled pollination	Parthenogenetic embryos	N/a	N/a	Eenink (1974a, b; 1975)
*Cucumis sativus*	Cultivar	Pollination with γ-irradiated pollen	Embryological analysis	Incomplete or failed transmission of the male genome	Globular parthenogenetic embryos	80–100% of the ovules	N/a	Le Deunff and Sauton (1994); Faris and Niemirowicz-Szczytt (1999)
*Malus* spp.	*Malus* × *domestica*	Pollination with γ-irradiated pollen	Embryological analysis; ploidy level determination in AE nuclei	Incomplete or failed transmission of the male genome	Poorly developed embryos (2.6–20%)	20–50% of the ovules	Multinuclear AE (25–200 nuclei)	James *et al.* (1985); Nicoll *et al.* (1987); Zhang and Lespinasse (1991)
*Monotropa uniflora*	N/a	Prevention from pollination; high temperature conditions; high osmotic pressure	Embryological analysis	Lack of pollination+ higher temperature	2-celled parthenogenetic embryos (rare)	N/a	N/a	Shibata (1902); based on: Frödin *et al.* (1921); Dahlgren (1927)
*Nicotiana* sp.	N/a	Pollination with γ-irradiated pollen	Embryological analysis	Incomplete or failed transmission of the male genome	Parthenogenetic embryos (rare)	N/a	N/a	Musiał and Przywara (1999a)
*Prunus domestica*	Cultivar	Pollination with γ-irradiated pollen	Embryological analysis; ploidy level determination in AE nuclei	Incomplete or failed transmission of the male genome	Likely parthenogenetic embryos (up to 33%)	Up to 58% of the ovules	Multinuclear stage of AE	Peixe *et al.* (2000)
*Theobroma cacao*	Clones	Pollination with γ-irradiated pollen	Morphology (bean dissection)	Incomplete or failed transmission of the male genome	Abnormal heterozygous embryo development (~3–50%)	~100% of the ovules	Mature (cellular) stage of AE	Falque (1994)

N/a—not applicable or not available.

#### Spontaneous development of AE in natural and cultivated sexual plants

Historically, the occurrence of AE in sexual plants in nature have often arisen by accident in cases where botanists investigated the cause of clonal reproduction despite seed production, as in the case of Eurasian *Anemone*. Populations of *Anemone nemorosa* and *Anemone ranunculoides* from Leningrad can produce normal seeds allowing efficient germination (Yoffe, 1969). However, individual populations from France and Poland produced a small portion of seeds which did not germinate. Embryological analysis indicated that those seeds contained an underdeveloped embryo and endosperm, or only the endosperm. The ovules went through a single fertilization event of the egg cell, whereas the diploid central cell developed autonomously (Trela, 1963a, b; Brouland, 1968; Trela-Sawicka, 1974). These observations were found in most of the ovules analysed and confirmed experimentally by flower emasculation and hand pollination. Even though embryo development was initiated, seed development was eventually inhibited and aborted due to disturbed mitoses during AE development (Trela, 1963b). This abnormal AE development is manifested primarily by the inhibition of cytokinesis and formation of restitution nuclei and, finally, nuclei of variable ploidy. Improper seed development of *A. nemorosa* and *A. ranunculoides* seems to be attributable to self-incompatibility as pollen tubes may not grow into the central cell (Trela-Sawicka, 1974; Fig. 1B.v).

Apart from *Anemone*, other examples have been reported (Table 1). In unpollinated *Juglans regia* ovaries (Tadeo *et al.*, 1994), the two polar nuclei fuse within 5 days after emasculation, leading to a 2n endosperm that starts cellularization (wall formation) about 5 days later. Both fertilized and unfertilized ovaries were examined for gibberellins (GAs), the plant hormone group that, along with auxin and cytokinin, controls the major aspects of plant growth, inter alia, flowering time and fertilization (Schwechheimer, 2012). Analyses of endogenous concentrations of GAs showed clear differences related to ovary pollination status (i.e. pollinated = high level versus unpollinated = low level) and the time elapsed since pollination. The lowest level of GAs correlated with the acceleration of cellularization of an autonomously developing homodiploid endosperm. Furthermore, multinuclear structures have been observed in unfertilized megagametophytes in tomato (*Lycopersicon esculentum*; Adamowicz *et al.*, 2000). The origin of such developing AE might be the division of the secondary nucleus or polar nuclei, or as a result of supernumerary mitoses (additional mitoses during embryo sac development) leading to the formation of multinuclear, abnormal, and mature gametophytes.

*In planta* divisions of nuclei in the unfertilized central cell have also been observed under controlled conditions in Arabidopsis Col-0 WT plants (Rojek *et al.*, 2021b). Thus, *in planta* AE development in sexual plants may show a similarity to native apomictic plants; however, seeds with only homodiploid endosperm are usually aborted unless parthenogenesis of the egg cell occurs (as in *Brassica oleracea*; Eenink, 1974a, b).

#### Experimental evaluation of AE *in planta* by modifying growth conditions

Induction of AE formation was experimentally tested and evaluated demonstrating that it can be induced by temperature changes or after inefficient pollination, e.g. after pollination of irradiated (with gamma-rays) pollen or delayed/ prickled (i.e. after interspecific or intergeneric) pollination (Eenink, 1974a, b; Musiał and Przywara, 1998; Table 2; Fig. 1B).

In 1902, Shibata reported the induction of autonomous development of the central cell in the amphimictic plant *Monotropa uniflora*, under high-temperature treatments (Shibata, 1902). In *Brassica* sp., diploid or haploid parthenogenesis is accompanied by AE development (Eenink, 1974a). In *B. oleracea*, after prickle pollination (often also delayed pollination, Eenink, 1974a, b, 1975), matromorphic seeds (i.e. with parthenogenetic embryo) contain AE. Doubled haploid lines of *Brassica napus* although rare, also form AE which accompanies the parthenogenetic embryo (Woźny and Rojek, 2020).

*In planta* pollination with irradiated pollen is a widely used technique to induce haploids in cultivated plants (e.g. apple, cucumber, kiwi, pear, rose, melon, citrus; Musiał and Przywara, 1998; Marin-Montes *et al.*, 2022), yet the percentage of haploids obtained is generally low. When *in vitro* culture is used after pollination with irradiated pollen, the efficiency of haploid production increases [see Musiał and Przywara (1998) and references cited therein].

Although irradiated pollen has been used for many years (first used in 1922 by Blakeslee *et al*.,1922), little information is available about fertilization and the early stages of embryogenesis after pollination with irradiated pollen. Only a few embryological studies have been carried out for the embryo of *Actinidia deliciosa*, *Pyrus*, *Prunus*, and *Cucumis*, and the endosperm is generally ignored (Table 2; see Musiał and Przywara, 1998; Faris and Niemirowicz-Szczytt, 1999; Peixe *et al.*, 2000).

The use of irradiated pollen has demonstrated that (i) irradiation interrupts or prevents double fertilization primarily by the inhibition of pollen tube growth and pollen viability (Fig. 1B.iii and iv); (ii) embryo (via parthenogenesis) and AE development is irradiation dose dependent; a low dose triggers mutational damage (e.g. Sanders *et al.*, 1991), whereas higher doses increase the frequency of parthenogenetic embryos. The parthenogenic effect of higher doses is commonly explained by the ‘Hertwig effect’. The phenomenon was found by O. Hertwig, in 1911, in frog spermatozoa which were exposed to ionizing radiation and then used to fertilize normal eggs. Hertwig observed a paradoxical situation in which increasing the dosage led to an increasing amount of embryonic death and abnormality, but the higher dosage led to the production of apparently normal offspring. It was explained in such a way that lower doses affect both the penetration of spermatozoa to the egg and syngamy, whereas higher doses affect (disrupt) penetration only, yet the egg is stimulated to gynogenetic development (parthenogenesis). Subsequent works confirmed both the observation and the explanation in animals and plants [Pandey and Phung (1982), and references cited therein]; (iii) with the higher radiation doses (>200 Gy), embryo and endosperm development is delayed in relation to control plants, although this effect of the applied dose is species dependent; (iv) AE development is manifested by the lack of cellularization, as in *Prunus* (Peixe *et al.*, 2000); (v) depending on the irradiation dose, various types of autonomous seeds can be obtained: normal, empty, with the presence of endosperm, embryo only, or with both embryo and endosperm (Fig. 1B.iii and iv); (vi) in general, AE contains fewer reserve substances compared with the wild-type endosperm; the amount of starch and lipids in the diploid endosperm is comparable to that in the central cell (e.g. Musiał and Przywara, 1998); (vii) AE nuclei may show karyotypic variability (polyploid nuclei are formed after disturbed mitotic divisions or endoreplication processes), as observed in apple trees (Nicoll *et al.*, 1987), whereas in other cases no such variability was observed, e.g. in kiwi ( Musiał and Przywara, 1999b); (viii) the synchronization of endosperm nuclei divisions may be disturbed.

To summarize, genetic, epigenetic, and biochemical signals can trigger AE in cases where the pollen grain carries non-functional genetic material. AE obtained via the aforementioned strategies may provide potential opportunities for studying homodiploid endosperm development using novel techniques (e.g. reporter analysis; Chen *et al.*, 2022).

#### Gaining insight into AE through the analysis of mutations and (epi)genomics

##### Mutant analyses.

In the past two decades, genetic analyses of *Polycomb Group Protein* (*PcG*) genes have revealed their significant role in the regulation of endosperm development. Characterization of mutation in three different loci in Arabidopsis: *FERTILIZATION INDEPENDENT ENDOSPERM* (*FIE*; Ohad *et al.*, 1996; 1999), *FIS2* (Luo *et al.*, 1999), and *MEDEA* (*MEA*; Grossniklaus *et al.*, 1998; Kiyosue *et al.*, 1999), collectively named the ‘*FIS* genes’, revealed that mutations of each of these genes, when maternally inherited, cause endosperm overproliferation (Hsieh *et al.*, 2003; Table 3). One of the variants of the Polycomb complex in Arabidopsis, FIS-PRC2, containing MEA, FIE, FIS2, and MSI1, controls cell proliferation during sexual reproduction (Derkacheva and Hennig, 2014; Mozgova and Henning, 2015). Mutations that impair the functions of this complex result in phenotypic changes both before and after fertilization and cause abnormal cell proliferation in Arabidopsis (Guitton and Berger, 2005; Leroy *et al.*, 2007; Zhang *et al.*, 2018) and rice (Li *et al.*, 2014). In the *mea*, *fie*, *fis2*, *and msi1* mutants, the central cell initiates endosperm development in the absence of fertilization (Fig. 1C); when fertilization occurs, the embryo and endosperm derived from mutant female gametes show developmental abnormalities. It is worth noting that FIE {a WD-40 protein that is homologous to the *Drosophila* enhancer of zeste [E(z)]} is present in all known PcG complexes in Arabidopsis. It is encoded by a single (unique) gene and interacts with each of the components of the complex which is demonstrated by the lack of formation of these complexes (PCR2-like complexes) in *fie* mutants [Oliva *et al.* (2016) and references cited therein].

**Table 3.** Occurrence of AE in sexual angiosperms *in planta* in Arabidopsis and rice mutants.

Taxon	Population or genotype	Experimental regime	Method	Added Factor(s)	Embryo companion	Occurrence (frequency)	Advancement in the development	References
	***In planta* in *Arabidopsis and rice mutants***							
*Arabidopsis thaliana*	*bga-1/BGA* *(borgia)*	γ-irradiation of seeds; emasculation and prevention from pollination	Morphology (ovule enlargement)	Gametophytic maternal effect of mutation	N/a	12.8% of the ovules	N/a	Guitton *et al.* (2004)
*Arabidopsis thaliana*	*emb173/EMB* *embryo-defective173*	T-DNA insertion; emasculation and prevention from pollination	Embryological analysis	Gametophytic maternal effect of mutation	No	1.8% [*emb173/+* (WS)]	Multinuclear stage of AE	Kiyosue *et al.* (1999)
	*eml1–2 eml3–4* *emsy-like (eml) histone readers*	T-DNA insertion; emasculation and prevention from pollination	Embryological analysis	Elevated auxin signalling and transport pathways around fertilization	Parthenogenetic embryos (2%)	8.3% of the ovules	Underdeveloped AE	Milutinovic *et al.* (2019)
	*DD65::TAA1; DD65::YUC6*	Transgenic lines; emasculation and prevention from pollination	Embryological analysis	Ectopic auxin production in the central cell	No	Up to 25% of the ovules	Several to multinuclear stage of AE	Figueiredo *et al.* (2015)
	*fie (fis3; fie-1/FIE); fertilization independent endosperm*	Mutagenization of homozygous *pop1* seeds with ethylmethanesulfonate; emasculation and prevention from pollination	Embryological analysis	Gametophytic maternal effect of mutation	No	47% [*fie-1/+* (Ler)] 38.5% (*fie-11/+*) of the ovules	Multinuclear stage of AE	Ohad *et al.*(1996, 1999); Kiyosue *et al.* (1999)
	*fie-1/FIE; MET1 a/s* *METHYLTRANSFERASE I* antisense	*fie1* and reduced methylation; emasculation and prevention from pollination	Embryological analysis	Gametophytic maternal effect of mutation; reduced DNA methylation	No	51% of the ovules	Multinuclear and cellularization stages of AE	Vinkenoog *et al.* (2000)
	*fis2; fis2–7* *fertilization independent seed 2*	Mutagenesis (gamma rays); emasculation and prevention from pollination	Embryological analysis	Gametophytic maternal effect of mutation	Embryo-like structure (a few cases); no (*fis2–7)*	N/a; 60% (*fis2-7/+)* 31.7% (*fis2-6/+*) of the ovules	Multinuclear (*fis2–7*) and cellularized stages of AE	Chaudhury *et al.* (1997); Guitton *et al.* (2004)
	*mea (medea; fis1)* *mea/f*644	Mutagenesis (gamma rays); emasculation and prevention from pollination	Embryological analysis	Gametophytic maternal effect of mutation	No	20.6% *mea-6/+* (Ler) 7.5% *f*644/+ (Ler) 18.6% *f*644/*f*644 (Ler) 10.3% *f*644/+ (Col) 1.5% *f*644/+ (WS)	Multinuclear AE	Grossniklaus *et al.* (1998); Kiyosue *et al.* (1999); Guitton *et al.* (2004)
	*mea* ^*-/-*^ *× cdka;1* ^*+/-*^ (*cdka;1, A-type cyclin-dependent kinase*)	Controlled pollination with *cdka;1*^*+/-*^ pollen	Embryological analysis; ploidy level determination in AE nuclei	*mea* gametophytic effect together with single sperm cell with mutation in the key cell cycle regulator CDKA;1	Full embryo development after single fertilization	~20% of the ovules	Full AE development—until cellular stage	Nowack *et al.* (2006, 2007); Ungru *et al.* (2008)
	*msi1* (*multicopy suppressor of ira1)*	Mutagenesis (gamma rays); emasculation and prevention from pollination	Embryological analysis	Gametophytic maternal effect of mutation	Parthenogenetic embryo; no (*msi1–2*)	90% (*msi1-2*) of the ovules	Multinuclear stage of AE	Köhler *et al.* (2003a); Guitton *et al.* (2004); Guitton and Berger (2005)
	*MET1/met1–-9* *met1–9/met1–9* *cytosine methyltransferase MET1*	Knock-out mutation; emasculation and prevention from pollination	Embryological analysis	Lack of MET1 (disruption in silencing of *FWA* paternal allele in the endosperm)	No	3–7% of the ovules	Few-nuclear	Rojek (2010)
	*rbr1 (retinoblastoma related 1)*	T-DNA insertion; emasculation and prevention from pollination	Embryological analysis	Mutation	No	27.3% of the ovules	Syncytial (multinuclear) stage	Ebel *et al.* (2004)
	*rgtb1* *(RAB geranylgeranyl transferase beta-subunit 1)*	T-DNA insertion; emasculation and prevention from pollination	Embryological analysis; auxin detection in AE	Interfering with vesicle traffic that affects PIN1 recycling/auxin efflux alternation	No	10% of the ovules	From few to multinuclear stage of AE	Rojek *et al.* (2021b)
	WT *× cdka;1*^*+/-*^ (*cdka;1, A-type cyclin-dependent kinase)*, 14 accessions	Controlled pollination with *cdka;1*^*+/-*^ pollen	Embryological analysis; number of division cycles; seed growth parameters; QTL analysis	Single sperm cell with mutation in the key cell cycle regulator CDKA;1	Underdeveloped embryo after single fertilization	~50% of the ovules in each ovary	1–48 nuclei of AE	Ungru *et al.* (2008)
*Oryza sativa*	*emf2a* *(embryonic flower2a)*	CRISPR/Cas9 edition; emasculation	Embryological analysis	CRISPR/Cas9 edition	No	36.8 ± 7.3% of the ovaries	NCDs stage of multinuclear AE	Tonosaki *et al.* (2021)
	*OsFIE2 RNAi lines*	RNA interference	Embryological analysis	*OsFIE2*-silence	No	~12% of the ovaries	Multinuclear to incomplete cellularization stage of AE	Li *et al.* (2014)

N/a—not applicable or not available.

Research on *FIS-PRC2* genes indicates that fertilization of the central cell is not necessary to stimulate endosperm formation, even in the early stages of development, and also that the pathways of egg and central cell development are independent, as the *fie* mutation does not initiate embryogenesis in the absence of fertilization (Ohad *et al.*, 1996). In both non-pollinated and pollinated *msi1* mutant ovules of Arabidopsis, the diploid endosperm develops without fertilization from an unfertilized central cell. On the other hand, the development of an embryo or embryo-like structure, although disturbed, must be preceded by fertilization (Köhler *et al.*, 2003a). An additional case has been reported in rice (*Oryza sativa*), where a mutation in the *EMBRYONIC FLOWER2a* (*OsEMF2a*) gene, encoding a zinc-finger protein which is a member of the PRC2 complex, induces AE in the absence of fertilization. Importantly, although fertilization takes place in *OsEMF2a* mutant plants, the developmental transition from one stage to another during endosperm development is delayed, demonstrating that OsEMF2a-containing PRC2 controls endosperm developmental programmes before and after fertilization (Tonosaki *et al.*, 2021). Maternal *BABY BOOM (BBM*), a key gene required for zygotic embryogenesis that is expressed mainly paternally (Khanday *et al.*, 2019), has also been shown to induce parthenogenesis and AE proliferation when it is overexpressed maternally in Arabidopsis (Chen *et al.*, 2022). When *bbm plt2* mutant ovules were fertilized, the endosperm failed to cellularize, similarly to the state of endosperm development in *fis-PRC2* mutants. Interestingly, BBM directly targets the *FIE* promoter, one of the FIS-PRC2 subunits (M. Li *et al.*, 2022); thus, BBM and PLT2 might redundantly regulate *FIE* expression during early endosperm development.

AE formed in *fis-PRC2* mutants after self-fertilization or without fertilization, shows limited development, and AE cellularization is rare in *mea* and *fis2* mutants (Chaudhury *et al.*, 1997). This phenotype is improved when the *fie* mutant of Arabidopsis is crossed with a mutant leading to reduced levels of genome methylation [*fie-1*/*FIE*; *MET1* (methyltransferase) a/s] in which endosperm development proceeded further and cellularized in the absence of fertilization (Vinkenoog *et al.*, 2000; Vinkenoog and Scott, 2001; Fig. 1C.iv). A similar effect has been observed in *mea* ovules, which are fertilized by pollen deficient in cyclin-dependent kinase A;1 (CDKA;1), resulting in normal endosperm development (Nowack *et al.*, 2007; Fig. 1C.iii). *cdka;1* mutant pollen contains only a single haploid gamete that exclusively fertilizes the egg cell, leaving the central cell unfertilized. The central cell in these ovules autonomously undergoes a few rounds of free-nuclear divisions (Nowack *et al.*, 2006) sufficient to induce the completion of seed development (Nowack *et al.*, 2007).

Both experiments underline the specific role of *FIS-PRC2* (histone methylation) and MET1 (DNA methylation) in genomic imprinting, i.e. an epigenetic phenomenon leading to parentally biased gene expression (Batista and Köhler, 2020). FIS-PRC2 via incorporating histone methylation, leading to H3K27me3, is responsible for the silencing of the maternal alleles of paternally expressed genes (PEGs) in the central cell before fertilization, and sustains this silencing in early endosperm after fertilization (Moreno-Romero *et al.*, 2019). In the case of MET1, it is active in the sperm cells and constitutively marks both maternally expressed genes (MEGs) and PEGs with DNA methylation. Although MEGs are probably silenced, PEGs keep transcriptional activity because DNA methylation marks prevent the deposition of H3K27me3 by FIS-PRC2. After fertilization, the presence of these DNA methylation marks in paternal alleles still prevents the deposition of the methyl group required for the formation of H3K27me3, thus allowing for the transcription of this allele. Maternal expression in the endosperm requires the removal of maternal DNA methylation which is achieved by DNA glycosylase DEMETER (DME, according to Batista and Köhler, 2020).

Thus, when fertilization takes place, the absence of a paternal genome in seeds derived from a *fis* × *cdka;1* cross is compensated by the activation of maternal PEG alleles in the FIS-PRC2-lacking endosperm (Nowack *et al.*, 2007). Fertilization of the *fis*-class central cell by hypomethylated pollen can drive full development of the endosperm due to reactivation of the maternal alleles of the PEGs (Vinkenoog *et al.*, 2000).

In line with these observations, *fis*-class mutants initiate autonomous (yet not full) endosperm development in unfertilized ovules probably due to the reactivation of maternal PEG alleles that ectopically trigger seed developmental pathways, bypassing the contribution of the paternal genome.

Summarizing the above observations on the *fis*-class mutation, the basic assumption is that these mutants have (epi)genetically established the ability to trigger in part development of AE, or even full development, if it is accompanied by hypomethylation (only AE in the lack of fertilization) or *cdka;1* mutation (embryo and endosperm development after single fertilization of the egg cell).

The *cdka;1* mutation has turned out to be a useful tool for studying embryo-endosperm dependency and has strikingly contributed to significant knowledge on AE development. Ungru *et al.* (2008) conducted a detailed study on *cdka;1* single fertilization in several *A. thaliana* WT accessions. Their results showed that (i) all tested accessions initiate and develop AE at different rates; (ii) single-nucleated AE (i.e. substantially, an unfertilized central cell in the ovule with fertilized egg cell) appears to be functional, and lead to the differentiation of the central cell into endosperm along with morphological changes in the single-fertilized ovule which are independent of cell divisions. In seeds with a single endosperm nucleus differentiation of the endothelium layer was induced; (ii) embryo develops, even if the central cell nucleus remains undivided; (iii) endosperm proliferation in WT × *cdka;1* seeds ceased ~3 days after pollination (DAP). Independently of this, the embryo continued to grow for three more days, reaching on average a size ~50 cells at 9 DAP; (iv) embryo development is limited when no, or very little, endosperm is formed.

Thus, the presented data reveal the autonomy of embryo development, but also confirm that further embryo growth depends on the presence of a fully developed endosperm. Importantly in the context of AE, the developmental potential for the endosperm appears to be already programmed into the central cell as a part of the female gametophyte and neither fertilization nor proliferation of this cell is required for the adoption of this fate (according to Ungru *et al.*, 2008).

##### (Epi)genetics.

Unlike parthenogenesis, the genetic control of the AE component in apomixis has not been thoroughly investigated except in apomictic *Erigeron* and *Hieracium* (Noyes *et al.*, 2007; Ogawa *et al.*, 2013). This is partly because many well-studied apomicts, such as *Pennisetum*, are pseudogamous and lack AE. In other apomictic model plants, such as the *Boechera* genus, the penetrance of the phenotype is low (~15%; Aliyu *et al.*, 2010). An ideal model for uncovering the basis of AE could be *Taraxacum*, in which the complete penetrance of AE development exists. Such cases can be combined with the known pattern of endosperm formation, as well as the availability of specific mutations that can induce autonomous formation (Van Dijk *et al.*, 2020).

Several studies on sexual plants have shown that both alterations of histone H3K27 mediated by *PcG* genes, and DNA methylation via the *met1* mutation, give rise to AE *in planta* and *in vitro* (e.g. Ohad *et al.*, 1996; Vinkenoog *et al.*, 2000; Curtis and Grossniklaus, 2008; Rojek *et al.*, 2013, 2015).

Since mutation in the *FIE* gene leads to AE development in sexual Arabidopsis, its orthologue in *Hieracium* (named *Hieracium FIE* gene, *HFIE)* has been evaluated in sexual and apomictic *Hieracium* using RNAi-silencing (Rodrigues *et al.*, 2008). The results of these analyses showed that (i) RNAi silencing of *HFIE* does not induce AE initiation in sexual *Hieracium.* It results in failed endosperm cellularization and embryo arrest post-fertilization; and (ii) in apomictic *Hieracium* silenced *HFIE* results in both autonomous embryo and endosperm formation that are arrested at the globular embryo and nuclear endosperm stage. Based on these findings, it is evident that *HFIE* is required for endosperm development in both sexual and apomictic ovules and that HFIE acts via different mechanisms compared with strictly sexual Arabidopsis. Nevertheless, a separate genetic locus (*AutE*) is responsible for AE development in *Hieracium*, yet the gene regulating this phenomenon has not been identified to date (Ogawa *et al.*, 2013). Here, in two hybrid plants produced from crosses between sexual and apomictic *Hieracium*, 18% of the embryo sacs of sexual (i.e. meiotically derived) origin, developed endosperm autonomously (Ogawa *et al.*, 2013). Backcrosses made between AE lines with sexual species resulted in progeny with the AE phenotype.

Potentially non-coding RNAs are involved in the regulation of parthenogenesis and endosperm formation in certain apomicts such as *Paspalum simplex* (Galia *et al.*, 2019). The ORIGIN RECOGNITION COMPLEX (ORC) is a multiprotein complex that controls DNA replication and cell differentiation in eukaryotes. The homologue of subunit 3 of *ORC*-*PsORC3a* is specific for apomictic genotypes. *ORC*-*PsORC3a* is a pseudogene that is expressed at low levels constitutively in all developmental stages of apomictic flowers, whereas *PsORC3b*—the putative functional gene in sexual flowers—showed a precise time-related regulation (only at the anthesis and post-anthesis stages). The activity of *ORC3* in apomicts seems to be important in the formation of functional endosperm, with the ratio of maternal to paternal contributions differing from 2m:1p (Siena *et al.*, 2016). This finding also supports previous studies that in *Paspalum*, genome-wide DNA demethylation affects parthenogenesis, but not apomeiosis (Podio *et al.*, 2014; Galia *et al.*, 2019).

Methylation and aberrant genomic imprinting may also be crucial for other apomicts, such as eudicot and pseudogamous *Boechera*, yet empirical data in native apomicts are not yet available (Kirioukhova *et al.*, 2018). The paternally imprinted transcription factor *PHERES1 (PHE1*) promotes embryo growth. This gene is maternally repressed in female gametophytes of *A. thaliana* (Köhler *et al.*, 2003b). Unlike in Arabidopsis, the imprinted homologue of Arabidopsis *PHE1*-*PHEL1* in *Boechera* apomicts is maternally expressed, due to locus-specific changes in DNA methylation. This finding may indicate a role of reverse imprinting (i.e. alterations in the control of genomic imprinting) in the establishment of apomictic seed and also AE development. Epigenetic regulation of AE, as shown in *AtFIE/MEA* mutants, may take place also in triploid pseudogamous *Boechera* via *FIS2* and *FIE* gene orthologues (Yilmaz *et al.*, 2015). Two other genes, *APOLLO* (Corral *et al.*, 2013) and *UPGRADE 2* (Mau *et al.*, 2013), are differentially active in sexual and apomictic *Boechera* during ovule and seed development (Bakin *et al.*, 2022).

Analysis of the *FIE* gene demonstrated differences in the level of *FIE* methylation in Col-0 explants cultured on different media (Rojek *et al.*, 2015). At the start of the culture, DNA methylation in the gene was relatively low. In addition, analysis of histone H3K9 methylation showed a low level of methylation prior to fertilization. A hormone-free medium with a higher concentration of sugar seems to increase the methylation in the gene. Preliminary analyses of *FIE* methylation under stress *in vitro* indicated changes in methylation in recognition sites cut by *Hpa*II/*Msp*I enzymes. Considering the general reduction methylation in tissues *in vitro*, the *FIE* gene seems to exhibit relatively high activity in ovary tissues. *In vivo*, *FIE* activity is high before fertilization and just after fertilization. Perhaps its expression is regulated, as in the case of *MEA*, by antagonistically acting proteins, namely FIS-PRC2 (for H3K27me3 methylation), MET1 (for DNA methylation), and demethylase (DME) (Schmidt *et al.*, 2013). The altered methylation status of *FIE in vitro* probably affects the normal activity of FIS-PCR2 before fertilization (i.e. a silencing role in the central cell) and similarly to *fie*, allows endosperm to form independently. A fully matured AE generated by the application of steroid hormones *in vitro* could be attributable to a synergistic effect between histone modification and DNA methylation within a distinct set of common target AE genes (Schmidt *et al.*, 2013), thus urging further analysis of methylation of *FIE/ MEA in vitro* (Fig. 1D.iv).

In summary, AE formation in sexual and apomictic species seems to be controlled by a more complex genetic mechanism that functions independently from apomeiosis (suppressed meiosis) and parthenogenesis. It is likely that molecular mechanisms controlling the development of all components of the seed are more complex in different natural apomicts. In pseudogamous apomicts, endosperm development depends on the fertilization of the central cell, whereas parthenogenesis appears to remain repressed in the absence of fertilization as recently shown for *Boechera* (Schmidt *et al.*, 2014; Kirioukhova *et al.*, 2018; Binmöller *et al.*, 2022). Nevertheless, the alternation of the methylation status in the central cell by the FIS-PRC2 complex is probably the clue mechanism of AE initiation, at least in sexual plants.

##### Auxin signalling.

The importance of auxin and auxin-dependent genes in AE formation has been recently shown. For example, excess auxin in the sporophytic tissues of the ovule due to the *RAB geranylgeranyl transferase beta-subunit 1* (*rgtb1*) mutation in Arabidopsis plants increases AE formation in unfertilized ovules (from 2.8% in WT, to 10% in the *rgtb1*/*rgtb1* mutant) and influences embryo development in a maternal sporophytic manner (Rojek *et al.*, 2021b; Table 3). Endosperm formation requires high auxin levels in the central cell, which occur at the time of fertilization in Arabidopsis (Larsson *et al.*, 2017). Treatment of Arabidopsis flowers with exogenous auxin analogues [in particular 2,4-dichlorophenoxyacetic acid (2,4-D)] induces AE formation in a large proportion of WT ovules (Figueiredo *et al.*, 2015). Using the pDR5rev:3×Venus-NLS reporter construct for auxin detection, Rojek *et al.* (2021b) showed that in emasculated Arabidopsis WT flowers, the central cell nucleus frequently shows this reporter activity, revealing a detectable auxin response in the central cell before fertilization and multinuclear AE. Since the endosperm of flowering plants is characterized by genomic imprinting (Barlow and Bartolomei, 2014; Rodrigues and Zilberman, 2015; van Ekelenburg *et al.*, 2022), its development requires the activity of the paternal genome, and PEGs are the prime candidates for initiating endosperm development. Genes coding for auxin biosynthesis enzymes YUC10 and TAR1 are imprinted in the Arabidopsis endosperm (Gehring *et al.*, 2011; Hsieh *et al.*, 2011; Wolff *et al.*, 2011; Pignatta *et al.*, 2014; Figueiredo *et al.*, 2015).

Thus, auxin could be a key factor that drives endosperm development as the application of auxin to unfertilized ovules or ectopic production of auxin in the central cell is sufficient to trigger its replication and initiate endosperm development and appears to regulate endosperm cellularization in Arabidopsis (Rojek *et al.*, 2005, 2013, 2015, 2021b; Kapusta *et al.*, 2007; Figueiredo *et al.*, 2015; Fig. 1D).

#### Triggering full AE development *in vitro* by chemical treatment

In addition to disturbances or mutations affecting *in planta* AE development in sexual plants, other methods can trigger AE development, such as the use of *in vitro* culture of unfertilized ovules. Such techniques were used to investigate factors that may induce AE. Induction of AE *in vitro* has been observed in at least 19 species, with a special emphasis on *A. thaliana* (Table 4). The induction of AE *in vitro* has been reported in a number of other taxa (i.e., ornamental *Calendula officinalis* and *Rudbeckia bicolor*, and wild *Salix viminalis*), but the data remain unpublished and detailed information on AE development is only available from the authors. Nevertheless, data on AE from these species were summarized (Kuta *et al.*, 2009), which indicated that the induction of AE *in vitro* is by large species dependent.

**Table 4.** Occurrence of AE in sexual angiosperms *in vitro*.

Taxon	Population or genotype	Experimental regime	Method	Added Factor(s)	Embryo companion	Occurrence (frequency)	Advancement in the development	References
	***In vitro***							
*Allium cepa*	Inbred line	Emasculation; culture of unpollinated ovaries	Embryological analysis	10% sucrose; auxin; cytokinin	Parthenogenetic embryo (0.4%)	~3.6–6.5% of the ovules	4–16 nuclei (when accompanied by the embryo) nuclear or cellular (when AE only)	Musiał et al. (2001, 2005)
*Anemone ranunculoides*	Natural clonal population		Embryological analysis	6% sucrose	No	6% of the ovules	Few nuclear AE	Trzcińska (2007)
*Arabidopsis thaliana*	WT Col-0; La-0		Embryological analysis	10% glucose; 6% sucrose; auxin shock, auxin+cytokinin; steroid hormones; 5-azaC	Increased egg cell (mainly in the presence of steroid hormones)	1–26% of the ovules	From few to multinuclear stage of AE; NCDs and cellularization (in the presence of steroid hormones)	Rojek (2010); Rojek *et al.* (2005, 2013, 2015); Kapusta *et al.* (2007); Chen *et al.* (2022)
	*FIE/FIE* *FIE/fie-1*	Mutagenization of homozygous *pop1* seeds with ethylmethanesulfonate; emasculation; culture of unpollinated ovaries	Embryological analysis	6% sucrose; auxin+cytokinin; steroid hormones	Two cases of embryo-like structures	26–47% of the ovules	Multinuclear stage of AE; NCDs and cellularization (in the presence of steroid hormones)	Rojek (2010); Rojek *et al.* (2013, 2015)
	*MEA/MEA* *MEA/mea*	Mutagenesis (gamma rays); emasculation; culture of unpollinated ovaries	Embryological analysis	6% sucrose; auxin+cytokinin; steroid hormones	No	~8–17% of ovaries; 1–4% of ovules, dependent of the genotype and medium type	Few nuclear AE (*MEA/MEA*); multinuclear AE, NCDs stage- just before cellularization (*MEA/mea*)	Kałon (2013); Motyka (2013)
	*MET1/MET1* *MET1/met1*–*9* *met1*–*9/met1*–*9*	Knock-out mutation; emasculation; culture of unpollinated ovaries	Embryological analysis	6% sucrose; auxin+cytokinin; steroid hormones	No	3–9.3% of the ovules	From few to multinuclear stage of AE	Rojek (2010); Rojek *et al.* (2013, 2015)
	*EC1:AMV:BnBBM* *EC1:AMV:BnBBM-GR* *RPS5A:AMV:BnBBM*	Ectopic *BBM* expression in the egg cell, by CRISPR editions; emasculation; culture of unpollinated ovaries	Embryological analysis	10% glucose; *BBM* overexpression	Multicellular ectopic structures at the micropylar pole of the embryo (rare)	3.4–63.5% of the ovules	2–8 nuclear AE	Chen *et al.* (2022)
*Boechera stricta*	ES512; LTM accessions	Emasculation; culture of unpollinated ovaries	Embryological analysis	6% sucrose; auxin; nicotinamide; 5-azaC	Zygote-like structure (one case)	~1% of the ovules	Few to fewer nuclear AE	Fydryszewska (2016); Wodzak (2022)
*Brassica napus*	Cultivar		Embryological analysis	6% sucrose, auxin+ cytokinin; auxin shock	No	4–35% of the ovaries, 1–3 ovules per ovary	2–20 nuclear AE; tissue-like AE	Chmielowiec *et al.* (1997); Rojek (2000); Rojek *et al.* (2002)
*Capsella rubella*	Natural 2n ecotype from Monte Gargano, Italy (MTE)		Embryological analysis	5-azaC	No	~1% of the ovules	Few to fewer nuclear AE	Strzelec (2017)
*Capsella bursa-pastoris*	Pomeranian population		Embryological analysis	6% sucrose; auxin shock	No	3–6% of the ovaries	Few-nuclear AE; tissue-like AE	Trzcińska (2007)
*Gossypium hirsutum*	Cultivar	Emasculation; culture of unfertilized ovules	Embryological and ultrastructural analyses	Gibberellic acid +auxin	No	N/a	Limited number of free nuclear divisions in AE; precocious cell wall formation	Jensen *et al.* (1977)
*Helianthus annuus*	Cultivar	Emasculation; culture of unpollinated ovaries	Embryological analysis	6% sucrose	Haploid embryoids	N/a	N/a	Yang *et al.* (1986)
*Helleborus niger*	Population from the Botanical Garden (Poland)	Emasculation; (1) culture of unpollinated ovaries; (2) flowers under semi-vivo condition	Embryological analysis	(1) Auxin+ cytokinin; (2) starvation (only water) and higher temperature (27 °C)	No	(1) 50%; (2) 7% of the ovules	(1) 10–420 nuclei of AE; (2) partially cellular AE	Mól *et al.* (1995)
*Hordeum vulgare*	Cultivars	Emasculation; culture of unpollinated ovaries	Embryological analysis	Dark; higher temperature (25 °C); auxin+ cytokinin	No (gynogenic embryo and AE occurred under the same conditions but in different embryo sacs)	N/a	N/a	Huang *et al.* (1982)
*Lupinus luteus*	Population from the Botanical Garden (Poland)	Emasculation; culture of unpollinated ovaries	Embryological analysis	Auxin+cytokinin	No	10–20% of the ovules	4–85 nuclei of AE	Mól *et al.* (1995)
*Melandrium album*	Population from the Botanical Garden (Poland)	Emasculation; culture of unpollinated ovaries	Embryological analysis	Auxin+cytokinin	No	0.02% of the ovules	3–80 nuclei of AE	Mól (1992); Mól *et al.* (1995)
*Morus alba*	N/a	Emasculation; culture of unpollinated ovaries	Embryological analysis	Auxin+cytokinin	Gynogenic embryo	4% of the ovules	10–40 nuclei of AE	Thomas (2004)
*Oryza sativa*	Cultivars	Emasculation; culture of unpollinated ovaries	Embryological analysis	2-methyl-4-chlorophenoxyacetic acid (MPCA, synthetic auxin)	No (gynogenic embryo and AE occurred under the same conditions but in different embryo sacs)	‘In some ovules’	AE-like structure	Zhou and Yang (1981)
*Viola odorata*	Populations from Poland	Emasculation; culture of unfertilized ovules	Embryological analysis	Auxin; auxin+cytokinin	No	9% of the ovules	Few to multinucleate stage of AE; tissue-like AE	Wijowska *et al.* (1999a, b)
*Viola riviniana*	Populations from Poland		Embryological analysis	Auxin; auxin+cytokinin	Embryo-like structures in some ovules	N/a	Few to multinucleate stage of AE	Wijowska and Kuta (2000)
*Viola silvestris*	Populations from Poland		Embryological analysis	Auxin; auxin+cytokinin	No	N/a	Few to multinucleate stage of AE	Wijowska and Kuta (2000)
*Viola tricolor* (*Viola × wittrockiana*)	Commercial cultivar		Embryological analysis	Auxin; auxin+cytokinin	No	N/a	Few to multinucleate stage of AE	Wijowska and Kuta (2000)

N/a—not applicable or not available.

A basic medium enriched with a higher (5–10%) sucrose or glucose concentration is sufficient to initiate AE in *B. napus* (Rojek *et al.*, 2002) and several Arabidopsis genotypes (Rojek *et al.*, 2005, 2013; Kapusta *et al*., 2007; Chen *et al.*, 2022). Outside the *Brassicaceae*, only *Helleborus* AE is initiated under water supplemented with a high sucrose concentration and higher temperature (27 °C). Results from Arabidopsis and other species (see Table 4) indicate that the exogenous addition of hormones increases the frequency of AE induction and accelerates its development. The dose and the type of hormones are genus/species dependent. However, higher efficiency of AE development is usually achieved in the presence of auxin (Tables 3, 4). Induction of AE *in vitro* commonly originates from the mature central cell [Mól (1999) and references cited therein].

Frequency and advancement in the development of AE *in vitro* are strongly dependent on the genotype and the medium, at least in Arabidopsis. Despite the high frequency of AE development *in vitro*, e.g. in WT ovules and transgenic *EC1:AMV:BBM* ovules which ectopically express *BBM* in the egg cell (26% and up to 63.5%, respectively; see Chen *et al.*, 2022 for details), only a few nuclear (two to eight nuclei) syncytial AE were observed (Table 4). The Arabidopsis Col-0 WT showed the highest frequency of AE both on media without the addition of hormones but with sucrose and glucose (26.2%; Chen *et al.*, 2022) and on media with the addition of auxin. Furthermore, AE frequency was significantly higher when a concentration of 5-azacytidine (5-azaC) as low as 50 μM was applied, an inhibitor of DNA methyltransferases (Pecinka and Liu, 2014; up to 7.2% ovules; Rojek *et al.*, 2015). In addition, the application of epibrassinolide or mammalian sex hormone improved the frequency of AE development in *fie-1* mutant ovules (Rojek *et al.*, 2015).

Unfertilized ovules from heterozygous *FIE/fie* mutants form AE at much lower frequencies than expected (9.6–26.5% of ovules analysed, 3.5–6.2 ovules per ovary; Ohad *et al.*, 1996, 1999; Rojek *et al.*, 2013, 2015). Generally, *in vitro* culture is highly disruptive for inoculated ovules, and a significant decrease in the viable ovule number during culture was reported (e.g. Mól, 1999; Wijowska *et al.*, 1999a, b; Musiał *et al.*, 2005). Following the treatment, ovules continued developing during the first few (1–3) days, but then they shrunk due to stress conditions, thus leading also to the loss of the ovules in the *fie* mutation background (= ovules that developed AE). Interestingly, under the same (destructive) *in vitro* conditions, similar AE frequencies were observed in WT, *FIE/FIE*, and *FIE/fie* mutants cultured on hormone-free MS but enriched with 6% sucrose. These results suggest that (i) *in vitro* stress acts through *FIE* and serves as a trigger for AE in the central cell regardless of the mutation/genotype, (ii) AE may form *in vitro* in segregating WT female gametophytes in heterozygous *fie* mutants, and (iii) *in vitro* stress may enhance maternal sporophytic effects in heterozygous *fie* offspring and so in non-mutated ovules, i.e. ovules from the same ovary that do not carry the *fie* mutant allele, may follow the *fie* (= AE) path leading to a similar phenotype (Rojek *et al.*, 2013, 2015).

Several genes have been shown to take part in the regulation of AE development in Arabidopsis. In the case of *fie* mutants, this results from alterations in histone methylation patterns (= imprinting) during seed development. In addition, other genes were shown to take part in AE development as in the case of hypomethylation (full AE in *fie-1/FIE;MET1 a/s* ovules; Vinkenoog *et al.*, 2000) and the *cdka;1* mutation (AE development after a single fertilization; Ungru *et al.*, 2008; see also *Mutant analyses* section). Mutation in the *methyltransferase 1* (*MET1*) gene leads to lack of silencing of the *FLOWERING WAGENINGEN* (Julien *et al.*, 2006) paternal allele in the endosperm. Mutated plants show developmental changes associated with impaired expression of this flowering regulator. In *MET1/met1* heterozygotes flowering time is impaired, whereas *met1/met1* homozygotes showed significant delay in flowering time compared to WT (FitzGerald *et al.*, 2008; Rojek, 2010; https://www.arabidopsis.org/servlets/TairObject?type=stock&id=289612).

In contrast to *fie*, the *met1* mutation per se does not trigger AE (Vinkenoog *et al.*, 2000; FitzGerald *et al.*, 2008) and only a few cases of AE were observed *in planta* (Rojek, 2010; Table 3), similarly to wild-type Arabidopsis (Table 1; Figueiredo *et al.*, 2015; Rojek *et al.*, 2021b). Surprisingly, all variants of *met1* genotypes (*MET1/MET1*; *MET1/met1*, *met1/met1*) developed AE *in vitro* (Rojek *et al.*, 2013; Fig.1D.ii). Hence, stress conditions imposed *in vitro* act in part as a trigger similar to the phenotype of hypomethylated mutants *in planta*: *fie-1/FIE;MET1a/s/MET1a/s* and *mea-1/MEA;met1-3/MET1* (with AE but without a developing embryo, Vinkenoog *et al*., 2000; Schmidt *et al.*, 2013; Fig.1C.iv).

Collectively, the results obtained from *in vitro* culture of *fie* and *met1* mutants support the idea that the combination of culture conditions, genotype, and specific hormones may affect genomic imprinting, altering the activity of imprinted paternally (PEG) or maternally (MEG) expressed genes to some extent, and can lead to induction and even full development of AE (Fig. 1D.iii and iv). This finding is in accordance with several reports on epigenetic regulation (by FIS-PRC2 and MET1 interaction) of imprinted genes (Köhler *et al.*, 2012; Schmidt *et al.*, 2013). Nonetheless, how gene expression changes *in vitro* and which gene(s) are involved remains to be determined.

Interestingly, Mateo de Arias *et al.* (2020) demonstrated *in vitro* that the shifts from apomeiosis to meiosis or vice versa are metabolically regulated in *Boechera*. Apomeiosis switched to meiosis when premeiotic ovules of apomicts were cultured on media that increased oxidative stress (drought, starvation, and H_2_O_2_ applications). In contrast, meiosis switched to apomeiosis when premeiotic pistils of sexual plants were cultured on media that relieved oxidative stress (included antioxidants, glucose, abscisic acid, fluridone, and 5-azaC). The lattermost may also promote the shift from fertilization-dependent endosperm development to AE *in vitro*; thus, AE initiation may be metabolically regulated (Rojek *et al.*, 2015).

In summary, AE *in vitro* develops in parallel with the unfertilized yet intact egg cell, or the parthenogenetic embryo. In the absence of any other factors (hormones or chemical factors), *in vitro* conditions can trigger or at least facilitate autonomous development of the central cell since AE is present in both WT (control) and BBM overexpressed ovules *in vitro* (Rojek *et al.*, 2005, 2015; Chen *et al.*, 2022). Chemical factors, such as sugar and auxin alone or in combination with 5-azaC can trigger AE development *in vitro*, and steroid hormones are sufficient to allow the development of mature endosperm (cellular endosperm), at least in *A. thaliana* (Table 4; Fig. 1D).

## Structure and developmental patterns of AE formation

In apomicts, AE development was cytologically analysed in *Taraxacum officinale* (Van Dijk *et al.*, 2020; Underwood *et al.*, 2022 and references cited therein) and *Hieracium* (Koltunow *et al.*, 1998, Tucker *et al.*, 2001), as well as in some other taxa from the *Asteraceae*. In diploid and triploid apomictic *Boechera*, AE was examined using flow cytometry (Naumova *et al.*, 2001; Aliyu *et al.*, 2010; Voight-Zielinski *et al.*, 2012), and embryological analysis (Naumova *et al.*, 2001; Wodzak, 2022). In *T. officinale*, a high asynchrony of AE and embryo development was observed, with proper embryo formation but one-cell endosperm arrest, or the endosperm became multicellular prior to the division of the egg cell (Cooper and Brink, 1949). In *Hieracium*, AE is present as a homopolyploid structure in polyploids. The initiation of endosperm development is predominantly nuclear, similar to sexual plants, but in some seeds, the cellular endosperm is initiated (Koltunow *et al.*, 1998; Tucker *et al.*, 2003). Both, fertilization-induced and autonomous development of the endosperm in *Hieracium* differ in the spatial patterning of the early nuclear endosperm divisions. AE nuclei with the associated cytoplasm clumped together with irregular spacing between nuclei; however, this scenario normalized with increasing nuclear divisions, and during cellularization the endosperm resembled endosperm in sexual plants (according to Rodrigues *et al.*, 2008). Thus, AE expression in apomicts seems to be as variable as in their sexual counterparts.

Sexual AE *in planta* is a short-lived tissue that decays along with unfertilized ovules unless single fertilization or prickled pollination and/or mutation initiates the development of seeds or seed-like structures (Table 2; Fig. 1B).

In Arabidopsis, normal (i.e. after fertilization) endosperm develops along an axis from the anterior (micropylar) pole where the embryo is formed at the posterior (chalazal) pole where maternal nutrients transit. Three distinct regions are formed: the micropylar endosperm, the peripheral endosperm, and the chalazal endosperm (see Brown *et al.*, 1999; Li and Berger, 2012 for details). Development along this axis influences the orientation of syncytial divisions, the mitotic domains, and the migration of Nuclear Cytoplasmic Domains (NCDs) before cellularization. Cellularization occurs via the formation of the Radial Microtubule System (RMS), NCDs, and then alveolation (Olsen, 2004; Ali *et al.*, 2023 and references cited therein).

Since *FIS-PRC2* genes control the transition between developmental phases during endosperm development, *fie* mutants cannot reach the cellularization stage (Ingouff *et al.*, 2005; Weinhofer *et al.*, 2010). In Arabidopsis *fie*, *mea*/*f644*, and *msi1* mutants, the early stage of AE development is similar to endosperm development after fertilization (Ohad, 1996; Grossniklaus *et al.*, 1998; Kiyosue *et al.*, 1999; Guiton *et al.*, 2004). At the multinuclear stage, the AE has around 200 nuclei but lacks morphologically distinct chalazal endosperm characteristics, although clusters of nuclei have been found occasionally in a common cytoplasm at the periphery of the embryo sac (Ohad *et al.*, 1996; Vinkenoog *et al.*, 2000).

It is important to indicate that in *fis* mutants, seed development is impaired as a whole, when the female gametophyte carries the *fis* allele. Thus, the *fis*-mutation-induced altered endosperm phenotype, with significant overproliferation of the chalazal/posterior region, influencing post-fertilization endosperm development when pollination has occurred (Vinkenoog *et al.*, 2000; Sørensen *et al.*, 2001; Li and Berger, 2012). It is difficult to estimate whether this phenotype causes AE or rather endosperm formation after fertilization. Chaunchury *et al.* (1997) observed in some *mea* and *fis2* ovules with cellularized AE the formation of a zygote- or embryo-like structures; however, these structures did not develop beyond the endosperm cellularization stage before atrophying. Specific genetic combinations such as in the *mea*^*-/-*^*xcdka;1*^*-/+*^ mutants lead to the development of seeds bearing zygotic embryos and AE, where the homoparental and homodiploid endosperm was completely cellularized on day 6 after pollination (Nowack *et al.*, 2007).

Cellularization of AE can be triggered also in RNAi unfertilized ovules of rice *in planta* (Li *et al.*, 2014); however, cellularization is incomplete. Additionally, mutation in the rice *EMBRYONIC FLOWER2a (OsEMF2a*) gene is involved in the AE phenotype, influencing the proliferation of the central cell nuclei with separate cytoplasmic domains, storage compounds, starch granules, and protein bodies specific to the endosperm (Tonosaki *et al.*, 2021).

AE development observed *in vitro* is delayed when compared to the fertilized endosperm, and the developmental delay increases with the culture time. In the most examined species, *A. thaliana*, central cell division is delayed by 2 to 3 days and multinucleate stage formation is delayed by 5 to 6 days (Rojek *et al.*, 2005, 2013, 2015; Kapusta *et al*., 2007; Chen *et al.*, 2022). The mature female gametophyte, and the bi- or few-nucleate AE are predominant in 3 to 5-day-old ovules, and multinucleate AE functions till the ovules decay or are harvested (10–21 days; Rojek *et al.*, 2015).

Generally, AE development *in vitro* arrests at the nuclear stage with no signs of nuclei arrangement, similar to the stage before cellularization *in planta* (see detailed information in Tables 1–4). Following culturing of ovules, one can observe that in all developmental stages events in which the central cell nucleus form multinucleate AE (Rojek *et al.*, 2005, 2013). AE often over-proliferates at the chalazal/posterior pole, thus resembling the endosperm of *fis*-class mutant seeds (e.g. Guitton *et al.*, 2004) and also seeds produced by crosses of diploids with tetraploids/hexaploids (Adams *et al.*, 2000; Vinkenoog *et al.*, 2000).

Ploidy, nuclei size, and the number of nuclei also vary and depend on the genotype, stage of embryo sac development at inoculation, and *in vitro* conditions. AE nuclei can originate from the homodiploid central cell nucleus, or from unfused haploid polar nuclei (i.e. Polygonum-type of embryo sac).

In contrast to endosperm development *in vivo* (e.g. Brown *et al.*, 1999; Ali *et al.*, 2023), during *in vitro* development of multinucleate AE, a variety of developmental patterns are observed. In Arabidopsis, under *in vitro* conditions, the first steps of AE development are reminiscent of those observed *in planta* after fertilization, with a binucleated central cell. As observed by Rojek *et al.* (2013, 2015), multinucleate AE induced *in vitro* has the following characteristics: (i) AE nuclei with cytoplasm forming a structure resembling endosperm which develops *in vivo* just before cellularization (NCDs); (ii) AE nuclei decrease in size but increase in number; (iii) AE nuclei in various size form clusters in three distinct regions of the embryo sac, especially proliferating in the micropylar region and usually accompanied by an enlarged egg cell; (iv) AE nuclei connected with cytoplasm form a network; and finally (v) AE as a tissue fill the entire embryo sac (tissue-like AE; Rojek *et al.*, 2015), and resembles cellularized AE in *fis2* mutants (Chaunchury *et al.*, 1997).

The structure and frequency of the AE depend on the genotype or ecotype of the plant but in general, these differences concern only AE frequency or the time of development (advancement). Thus, Arabidopsis is an example with a high tendency to develop fertilization-independent endosperm. Interestingly, preliminary experiments on its close relative *Capsella rubella* (Strzelec, 2017), in addition to sexual and apomictic *Boechera* genotypes resulted in a low rate of AE induction when auxin and 5-azaC were used. The presence of a mature embryo sac was crucial for the success of AE induction besides variation of the genotype (Fydryszewska, 2016; Wodzak, 2022).

While examining the effect of mutations on the epigenetic machinery as in the case of *fie* ovules, AE is expected to be formed in a much higher frequency than in ovules from pistils not carrying the mutation. The *fie* mutation is lethal in homozygous plants (*fie1/fie1*); however, heterozygous ovaries *in vitro* contain ovules carrying a mutant (*fie*) or normal *FIE* allele. Four types of ovules were observed in each single ovary simultaneously, during *in vitro* culture of *FIE-1/fie-1* (Rojek *et al.*, 2013, 2015): first class, with AE at the early stage (1–10 nuclei; a few nuclear AE); second class, with over-proliferated AE is similar to AE in the *fie* mutant *in planta* (multinuclear AE); third class, where AE develops just before cellularization; and fourth class, which contain cellularized AE. The last two classes have never been reported in *fie* ovules *in planta* (Ohad *et al.*, 1996, 1999) and have arisen due to *in vitro* conditions (Fig. 1D.iii). The first two classes occurred in the average proportion of a few nuclear AE: multinuclear AE, i.e. 5%: 17.4% (Rojek *et al.*, 2013). This finding may indicate that AE can develop in WT ovules independently from fertilization. Moreover, AE can also be formed *in vitro* in hypomethylated mutants (e.g. *met1*) that do not produce AE *in planta* (Fig. 1D.ii).

The application of media with steroid hormones to isolated ovules *in vitro* is important to understand and further explore AE development beyond the phenotype that *fie* mutants present. Overcoming the limitation of the *fie* mutant (i.e. the lack of cellularization of AE) can facilitate the discovery of the key mechanisms that will enable full AE development from the nuclear stage to the cellular stage.

Commonly, the central cell develops into AE while subsequent degeneration takes place in the egg cell apparatus. However, several embryological reports showed that *in vitro* (i) synergids and the egg cell remain intact as long as the female gametophyte maintain a maturity state just before fertilization; (ii) synergids (one or both) start degeneration in the same manner as the synergids upon the pollen tube entering the female gametophyte (FG); (iii) the egg cell remains intact much longer, is often increased in size, and even develops further. These data suggest similar signalling from synergids both upon fertilization and in unfertilized FG *in vitro* (Jensen *et al.*, 1977; Kapusta *et al.*, 2007; Rojek *et al.*, 2013, 2015; Chen *et al.*, 2022; Wodzak, 2022).

*In vitro* conditions rarely stimulate the divisions of both the egg cell and the central cell in the same ovule. Both autonomous structures developed in *Allium cepa* (Musiał *et al.*, 2001), a species with a high gynogenic potential (i.e. haploid autonomous embryo formation *in vitro*). Very few embryo-like structures were observed *in vitro* in WT and *fie-1*/*FIE* Arabidopsis ovules (Rojek *et al.*, 2015), *Viola riviniana* (Wijowska and Kuta, 2000), and *Boechera stricta* (Wodzak, 2022). The proper and complete development of the embryo is strongly dependent on the cellularization stage of the endosperm and sucrose influx from the endosperm. Thus, the relatively high concentration of sucrose for *in vitro* applications (6%) and the addition of steroid hormones seem to be sufficient to induce the complete development of the endosperm, but are not sufficient to trigger the development of a parthenogenetic embryo (Xiong *et al.*, 2021).

## The potential use of *in vitro* AE induction in agriculture

So far, the advantage of apomixis, such as fixation of heterosis and other non-additive genetic traits, have not been successfully incorporated into major crops despite recent advances in understanding the mechanisms leading to apomixis in native plants (see Underwood *et al.*, 2022). Importantly, a functional balanced apomictic endosperm or AE has not been achieved in recent artificial apomictic crops, and as such other solutions are being attempted to save the obtained apomictic embryos.

Using AE in synthetic apomixis seems to be not mandatory since the 2:1 maternal-to-paternal genome ratio in the endosperm, which is required for the appropriate development, is maintained together with the formation of viable seeds in MiMe + BBM1 rice plants (Khanday *et al.*, 2019; Vernet *et al.*, 2022). Nevertheless, the formation of stable high levels of synthetic apomictic seeds is dependent on the fertilization of the central cell. Thus, engineering AE may be ideal to achieve a complete autonomous apomictic system.

Inducing AE in sexual plants is poorly studied and is a huge challenge due to the molecular and (epi)genetic complexity of AE development. Dicots and monocots differ in AE development, and the dynamics underlying this complex process rely on genome balance, epigenetic regulation, and parent-of-origin effects founded upon the contribution of the male gamete. In addition, further understanding of genetic modifier elements, protein interactions, and regulatory pathways underlying embryo-endosperm development is needed. Nevertheless, a functional analysis of *FIE* (e.g. in maize *ZmFIE1* and *ZmFIE2*; Danilevskaya *et al.*, 2003; Gutiérrez-Marcos *et al.*, 2006) and PcG mutants leading to AE development in Arabidopsis and rice (*OsFIE2*, Li *et al.*, 2014) is key for future genetic engineering. Genome editing and demethylase studies, which may allow for the activation of repressed (imprinted) female genes, may lead to the onset of AE. Such studies may provide more information on the epigenetic background linked to these genes and their contribution to AE.

The central role of auxin in initiating seed development indicates that it could also play a key role in AE development (e.g. Roszak and Köhler, 2011; Figueiredo *et al.*, 2015, 2016; Rojek *et al.*, 2021a, b). This idea is supported by studies that applied auxin to unfertilized ovules and the depletion of gametophytic FIS-PRC2 function which led to apomictic-like endosperm development in sexual *A. thaliana* (Chaudhury *et al.*, 1997; Ohad *et al.*, 1999; Köhler *et al.*, 2003a; Rojek *et al.*, 2005, 2013, 2015; Roszak and Köhler, 2011; Figueiredo *et al.*, 2015, 2016). Thus, a relationship between FIS-PRC2 function, auxin activity, and activation of AE development seems plausible. FIS-PRC2 is specific to the central cell and its descendent endosperm and contributes to the establishment of genomic imprinting (Mozgova *et al.*, 2015; Rodrigues and Zilberman, 2015; van Ekelenburg *et al.*, 2022). Currently, it has been demonstrated that (i) the development of autonomous seeds in *fis*-class mutants shows the activation of the PEG *YUC10* in the unfertilized central cell, which deploys auxin production and is normally repressed in the central cell but expressed only in the endosperm after fertilization; (ii) *fie* autonomous seeds show ectopic activation of auxin reporters, thus indicating fertilization-independent activation of auxin signalling; and (iii) exogenous application of auxin or ectopic auxin production in unfertilized ovules leads to a phenocopy of the *fie* phenotype (Figueiredo *et al.*, 2015; Rojek *et al.*, 2015, 2021b).

Altogether, these findings strongly support the hypothesis that autonomous seed development in *fis*-class mutants is linked to auxin production.

The primary limitation of all *fis-*class mutants or synthetic apomictic seeds is the lack of proper endosperm development and dependency on central cell fertilization. An alternative approach to AE production in autonomous seeds may derive from exploring the conditions under which AE can be fully developed. Successful and complete formation of AE *in vitro* in Arabidopsis, induced by the application of steroid hormones, is a new tool for testing this process in other plants (Rojek *et al.*, 2015). Although understanding of the regulatory functions of endogenous progesterone and androsterone in plant stress response remains at the physiological level, exogenously applied mammalian and plant steroid hormones induce AE cellularization (Rojek *et al.*, 2015; H. Li *et al.*, 2022). Since the role of abscisic acid (ABA) has been revealed in endosperm cellularization and uncellularized endosperm-mediated embryo arrest (Xu *et al.*, 2022), exogenously applied steroid hormones may substitute impaired ABA synthesis, contributing to signalling AE development. These conditions can be successfully transferred to *in planta* conditions (Figueiredo *et al.*, 2015). Testing the application of exogenous hormones *in planta* may allow for the complete development of AE. This may facilitate the ongoing research on fertilization-independent seed production in model plants and crops.

Following a century of research, recent discoveries have provided us with additional tools to attempt manipulating apomixis in sexual crops (e.g. Mau *et al.*, 2021; Chen *et al.*, 2022; Underwood *et al.*, 2022).

Although apomeiosis (in *Hieracium*, *Taraxacum*, *and Boechera*), and parthenogenesis (in *Hieracium* and *Taraxacum*) clearly possess major loci of apomixis, recent studies show the presence of a third critical locus, probably constituted by several smaller modifier genes that jointly induce AE. AE is still the least understood phenomenon in apomictic *Hieracium* and *Taraxacum*. A two-pronged strategy combining data from native apomicts and experimentally induced AE in sexual plants can provide useful information to improve the ability to develop AE in crops.

## Conclusion

Much knowledge has been gained in the field of plant reproduction, yet there is much to explore regarding a better understanding of AE development. Reports on this interesting phenomenon have been accumulating slowly, yet recent studies help to explain and prove the observations of previous reports describing AE development in sexual plants.

Studies presented in this review show that: (i) EBN is highly conserved and regulated by the parent-of-origin effect and imprinting, and changes in the EBN lead to seed abortion in the majority of sexual plants. However, some plants, especially autonomous apomicts, overcome EBN and produce AE (e.g. Köhler *et al.*, 2010; Van Dijk *et al.*, 2020); (ii) the unfertilized central cell has the potential to develop into endosperm, yet it is suppressed until fertilization, by several mechanisms recruiting FIS-PRC2, DNA methylation, transcription factors, and hormonal regulators (Hands *et al.*, 2016); (iii) AE development is triggered by altering FIS-PRC2 function in sexual plants (Ohad *et al.*, 1996); (iv) AE is easily induced in Arabidopsis by exogenous hormonal and chemical triggers, and can develop fully *in vitro* (Rojek *et al.*, 2015); and (v) the shifts from apomeiosis to meiosis or vice versa are metabolically regulated in *Boechera in vitro* (Mateo de Arias *et al.*, 2020).

Hence, the ability to produce AE may emerge from mutation accumulation which enables ‘escape’ from extinction, as is proposed for apomictic populations (Lovell *et al.*, 2017; Paczesniak *et al.*, 2022). With reference to this, the parthenogenetic development of the embryo may be the first step where AE develops as an adaptive consequence, potentially making a perfect apomict which does not require fertilization. On the other hand, the potential of the unfertilized central cell to form endosperm may be the same in sexual plants and apomicts, and though paused, it may be quickly launched when fertilization fails, as evidenced in *in vitro* studies.

Although endosperm does not transmit its DNA to the next generation it is possible that the ability to form AE from an unfertilized central cell is a remnant feature that was carried along evolution, as in the case of *Ginkgo* where fertilization is initiated very late, at the end of the cellular growth phase of the female gametophyte; Friedman, 2001). Endosperm is believed to develop at the expense of a reminiscence sister egg cell; thus, perhaps the molecular regulation controlling its proliferation depends on environmental and internal cues, which evolved through plant evolution in a ‘loss’ scenario, to ensure the development of the embryo even when the second fertilization event did not occur. Alternatively, this mechanism may allow an unsynchronized late fertilization of the egg cell to facilitate embryo nourishment.

Thus, studies of AE development in sexual plants could facilitate the understanding of the triggers and mechanisms of AE initiation and full development.
